# Enhanced Analytical Performance in CYFRA 21-1 Detection Using Lateral Flow Assay with Magnetic Bioconjugates: Integration and Comparison of Magnetic and Optical Registration

**DOI:** 10.3390/bios14120607

**Published:** 2024-12-11

**Authors:** Artemiy M. Skirda, Alexey V. Orlov, Juri A. Malkerov, Sergey L. Znoyko, Alexandra S. Rakitina, Petr I. Nikitin

**Affiliations:** 1 Prokhorov General Physics Institute of the Russian Academy of Sciences, 38 Vavilov Street, 119991 Moscow, Russia; artemskirda@mail.ru (A.M.S.); jurimalkerov@yandex.ru (J.A.M.); znoykos@yandex.ru (S.L.Z.); a.s.rakitina@nsc.gpi.ru (A.S.R.); 2 Moscow Center for Advanced Studies, Kulakova Str. 20, 123592 Moscow, Russia; 3 National Research Nuclear University MEPhI (Moscow Engineering Physics Institute), 31 Kashirskoe Shosse, 115409 Moscow, Russia

**Keywords:** lateral flow, immunochromatography, biosensing, immunoassay, nanoparticles, magnetic particles, point-of-care testing, tumor marker, CYFRA 21-1, cytokeratin 19 fragment, label-free

## Abstract

A novel approach to developing lateral flow assays (LFAs) for the detection of CYFRA 21-1 (cytokeratin 19 fragment, a molecular biomarker for epithelial-origin cancers) is proposed. Magnetic bioconjugates (MBCs) were employed in combination with advanced optical and magnetic tools to optimize assay conditions. The approach integrates such techniques as label-free spectral-phase interferometry, colorimetric detection, and ultrasensitive magnetometry using the magnetic particle quantification (MPQ) technique. For the first time in LFA applications, the MPQ-based and colorimetry-based detection methods were compared side by side, and superior analytical performance was demonstrated. The limit of detection (LOD) of 0.9 pg/mL was achieved using MPQ, and 2.9 pg/mL with optical detection. This study has demonstrated that MPQ provides elimination of signal saturation, higher sensitivity (slope of the calibration curve), and a 19-fold wider dynamic range of detected signals. Both optical and magnetic detection results are comparable to the best laboratory-based tests with the added benefits of a 20-min assay duration and the LFA format convenience. The assay effectiveness was validated in human serum and artificial saliva, and high recovery rates were observed. The proposed approach offers rapid and reliable detection of molecular biomarkers and holds significant potential for point-of-care diagnostics, particularly in resource-limited settings.

## 1. Introduction

CYFRA 21-1, a fragment of cytokeratin 19, serves as a molecular biomarker predominantly for cancers of epithelial origin [1,2,3,4]. It is widely used in the diagnosis and monitoring of various malignancies, in particular, non-small cell lung cancer, squamous cell carcinoma of the lung, and muscle-invasive bladder carcinoma [5,6]. Cytokeratin 19, a type I cytokeratin, is a component of the intermediate filaments in the cytoskeleton of epithelial cells [7,8]. The levels of its water-soluble fragments (including CYFRA 21-1 released due to cell death mechanisms such as apoptosis and necrosis) often correlate with disease progression and poor prognosis in a range of cancers [9,10,11,12]. Furthermore, CYFRA 21-1 is valuable for monitoring patients after tumor resection and may facilitate early detection of recurrences [13]. Although its measurement is most common in serum, CYFRA 21-1 can also be quantified in other biological fluids including urine and, potentially, saliva [14,15].

Currently, numerous analytical methods are available for detection of CYFRA 21-1 [16,17,18]. The commercially available test systems based on enzyme-linked immunosorbent assays (ELISAs), electrochemiluminescent immunoassays (ECLIAs), and immunoradiometric assays (IRMAs) are widely implemented in clinical practice and feature high specificity and sensitivity [19,20,21,22,23,24,25]. Recent advancements have introduced more sensitive techniques for CYFRA 21-1 detection including those based on multilayer structures, electrochemical impedance immunosensors, label-free electrochemical biosensors, and photoelectrochemical immunosensors [26,27,28,29]. These novel methods offer advantages of multiplexing, reducing the assay time, and even the potential for early cancer detection. Nevertheless, the development of rapid point-of-care (POC) approaches suitable for resource-limited settings remains one of the unresolved challenges in enhancing the screening in remote or underserved areas, where access to medical services is limited [30].

One of the most promising technologies for rapid POC diagnostic test systems is the lateral flow assay (LFA) [31,32,33]. The LFA has gained wide adoption in diagnostics due to its simplicity, cost-effectiveness, and rapid onsite delivery of results. Notably, over the past few years, the LFA has driven significant developments due to the enormous demand for POC tests during the COVID-19 pandemic [34]. Various detection methods are used in the LFA, including optical techniques such as visual interpretation, quantitative colorimetry, fluorescence measuring, and surface-enhanced Raman spectroscopy [35,36,37,38], as well as magnetic methods, which utilize magnetometry combined with magnetic particles as detectable labels [39,40,41,42,43,44,45,46].

However, for detecting tumor markers such as CYFRA 21-1, the LFA-based tests still face challenges and do not yet match the sensitivity and accuracy of the laboratory-based methods like the ELISA and ECLIA. One possible reason for that is the detection of LFA signals only from the surface of the analytical membrane so that the labels located deeper inside it are not registered. Moreover, optimization of LFA conditions remains a complex task that involves the fine-tuning of multiple parameters such as particle biofunctionalization, buffer composition, flow rate through the membrane, and others [47,48]. These optimizations are often addressed through a trial-and-error procedure that may not always result in the most efficient test conditions [49].

In this work, we propose a new approach to developing LFA-based methods for CYFRA 21-1 detection based on magnetic bioconjugates (MBCs) along with novel magnetic and optical tools for optimization of analysis conditions. We employ multiple techniques simultaneously: (i) an optical label-free method of the spectral-phase interferometry (SPI) to characterize the kinetic parameters of anti-CYFRA 21-1 antibodies and to optimize their arrangement within the test system; (ii) static and dynamic colorimetric detection with background illumination correction for evaluating test line intensity; and (iii) ultrasensitive magnetometry based on the magnetic particle quantification (MPQ) method [45,50], which enables the detection of magnetic nanomaterials throughout the test strip volume and their distribution across all its components. For the first time in relation to the LFA, we compare side by side the MPQ-based and colorimetry-based detection methods and demonstrate record-breaking analytical characteristics of LFA-based detection of CYFRA 21-1. As a result, we achieved a limit of detection (LOD) of 0.9 pg/mL, a dynamic range spanning four orders of magnitude, and a sensitivity of 0.66 (representing the slope of the calibration curve in its linear region, with units of [orders of signal change] × [orders of concentration change]^−1^). Finally, we demonstrated the applicability of the developed LFA approach for detecting CYFRA 21-1 both in human serum and artificial saliva.

## 2. Materials and Methods

### 2.1. Materials

The following reagents were used: commercial carboxyl-modified magnetic microspheres Estapor^®^ (polystyrene-encapsulated superparamagnetic iron oxide nanoparticles with ferrite content > 50%, lot No. FR180380073, Merck, Darmstadt, Germany); 1-ethyl-3-(3-dimethylaminopropyl)carbodiimide hydrochloride (EDC·HCl) and (3-aminopropyl)triethoxysilane (APTES), Triton X-100 (Sigma-Aldrich, St. Louis, MO, USA); 2-(N-morpholino)ethanesulfonic acid (MES) monohydrate (PanReac AppliChem, Darmstadt. Germany); phosphate-buffered saline (PBS, pH 7.4) (PanEco-Ltd., Moscow, Russia); clones XC10 and XC42 of anti-CYFRA 21-1 murine monoclonal antibodies (mAbs), rabbit anti-mouse antibody, positive calibrator of CYFRA 21-1, and positive calibration human serum (Xema-Medica Co. Ltd., Moscow, Russia); Unisart^®^ NC140 nitrocellulose membrane (Sartorius, Goettingen, Germany); polyvinyl chloride backing cards GL-187 (Lohmann, Neuwied, Germany); casein, dimethyl sulfoxide (DMSO), sulfuric acid, and hydrogen peroxide (RusHim, Moscow, Russia); succinic anhydride (Acros Organics, Geel, Belgium); artificial saliva (bioXtra, Seneffe, Belgium); bovine serum albumin (BSA) and negative calibration human serum (Dia-M, Moscow, Russia). The running buffer for LFA contained 0.1 M PBS, 1% BSA, 0.1% casein, and 0.1% Triton X-100.

### 2.2. Magnetic Bioconjugates Preparation

Magnetic bioconjugates were prepared using the carbodiimide method [51]. First, 3 μL of carboxylated magnetic particles from the stock solution were suspended in 100 μL of a 1% EDC solution in MES buffer. After a 15-min incubation, the particles were washed with water by magnetic separation and then resuspended in PBS containing varying amounts of antibodies (1, 3, 6, or 9 μg of antibodies in 100 μL of solution). The incubation period was 1 h. Next, 10 μL of a 10% BSA solution in PBS was added to block unreacted carboxyl groups and stabilize the colloid followed by an additional 15-min incubation. Then, the MBCs were washed with water and stored in a 1% BSA solution in PBS with 0.05% sodium azide as a preservative. The MBCs were stored at 4 °C until use.

### 2.3. Determination of Kinetic Properties of Antibodies with Spectral-Phase Interferometry

To characterize the kinetic properties of antibodies, biosensors based on the previously described spectral interferometric methods were employed [52,53,54]. The detection principle involves registration of the biolayer thickness on the surface of the glass sensor chip by measuring the phase changes in the interferometric pattern.

Sensor chips biofunctionalized with mAbs were obtained as follows. Cover glasses were first cleaned in piranha solution (a 1:3 mixture of H_2_SO_4_ and 30% H_2_O_2_). The glasses were then washed three times with ethanol and incubated overnight in a 3% *w*/*w* solution of APTES in 96.6% ethanol. Subsequently, the glasses were washed three times with DMSO and incubated in 40 mL of DMSO containing 75 mg of succinic anhydride for 2 h. The glasses were then washed again with DMSO and dried in a hot-air sterilizer at 50 °C. The carboxylated glasses were biofunctionalized with mAbs using the EDC conjugation method [55], and excess activated carboxyl groups were blocked with a 0.1 M Tris-HCl (pH 7.0) solution. The thus obtained sensor chips were stored at room temperature until use.

For kinetic measurements, an increase in the SPI sensorgram (reflecting changes in the biolayer thickness over time) was observed after introducing 100 µL of CYFRA 21-1 in 1% BSA PBS (10 µg/mL). After that, as buffer was passed through the system, a sensorgram decline, which corresponded to dissociation of immune complexes, was recorded.

The sensorgram fragments corresponding to the association phase were fitted using the following equation:

R(t) = R_max_·(1 − e^(−k^_on_^·C+k^_off_^)·t^), (1)

where R(t)—response signal at time t, R_max_—maximum response, k_on_—association rate constant, C—analyte concentration, and k_off_—dissociation rate constant.

For the dissociation phase, the sensorgram was fitted using the following equation:

R(t) = R_max_·e^−k^_off_^·t^. (2)

### 2.4. Lateral Flow Assay: Test Strip Fabrication and Assay Procedure

To prepare the LFA cards, a nitrocellulose assay membrane, an absorbent pad, a glass fiber sample pad, and a conjugate pad were glued onto a substrate with a 2 mm overlap. A test line (mAb XC42, 1 mg/mL, density—1 μL/cm) and a control line (rabbit anti-mouse antibody, 0.5 mg/mL, density—1 μL/cm) were dispensed onto the assay membrane. The cards were cut into 2.5 mm test strips and stored in a sealed bag with desiccant until use. The assay involved placing the test strip’s conjugate pad in a sample until migration of the solution along the strip was complete. Depending on the experiment, the samples were prepared using one of three matrices: running buffer (PBS BSA 1% Triton X-100 0.1%, and an appropriate amount of casein); human serum, diluted with running buffer at a ratio of 1:50; and artificial saliva, diluted with running buffer at a ratio of 1:10. No additional sample preparation procedures were applied.

### 2.5. Optical Detection of LFA Strips

**Figure 1:**
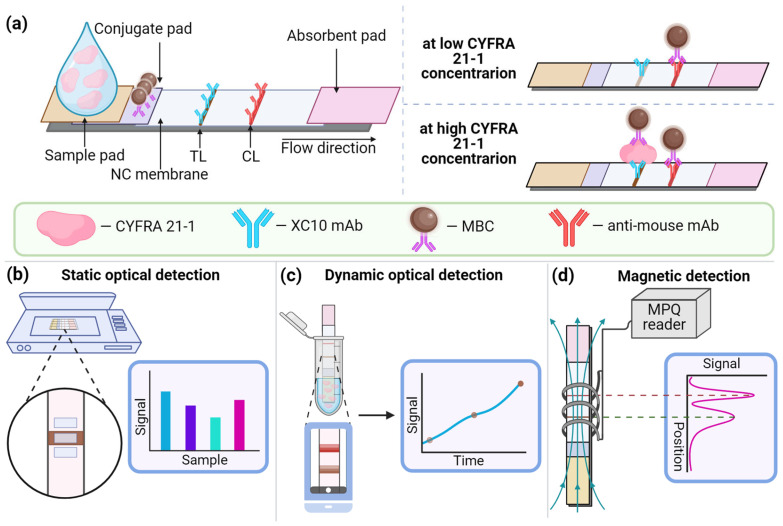
Detection of CYFRA 21-1 using a lateral flow assay based on magnetic bioconjugates: schematic of the test strip (**a**) and the application of various detection methods—static optical (**b**), dynamic optical (**c**), and electronic detection using the MPQ method (**d**).

Static optical signals were measured using a 16-bit Epson V550 office scanner at 600 DPI resolution (Figure 1b). The scanned images of the LFA test strips were processed using free and open-source ImageJ (v.1.54k) software [56] after pre-converting of the color images to grayscale. The optical signal was quantified as the absolute difference between the mean grayscale intensity within the region of interest (ROI) corresponding to the test line and the mean background intensity. The background intensity was calculated as the arithmetic mean of the signals measured at the identical-in-size ROIs located 3 mm down- and upstream of the analyzed test line. The dynamic signal acquisition involved recording the assay videos with a Samsung Galaxy A12 smartphone under stable illumination. The signal variations were analyzed using free and open-source ICY software (version 2.5.2.0) [57].

### 2.6. Magnetic Detection of LFA Strips

For magnetic detection, the LFA strips were placed in a magnetometer based on the magnetic particle quantification (MPQ) technique [50,58].

The measurement technique relies on detecting the nonlinear magnetic response of the sample when subjected to a two-component alternating magnetic field. The two components have amplitudes H_1_ and H_2_, and frequencies f_1_ and f_2_, respectively. Signal detection occurs at frequencies defined by f = 2 f_1_ ± f_2_. In our experiments, the magnetic field components were set as follows: H_1_ = 144 ± 10 Oe with frequency f_1_ = 152 Hz; H_2_ = 56 ± 5 Oe with frequency f_2_ = 156 kHz [45]. The detected “magnetic signal” represents the nonlinear response of the sample at these specific combination frequencies and is linearly proportional to the mass of magnetic nanoparticles present in the detection coil [58,59]. This approach allows for remote and non-destructive monitoring of the distribution and dynamics of MBCs in opaque objects [59]. The LFA strip was positioned so that the test line was centered within a cylindrical detection coil, and the magnetic signal was recorded for 10 sec. In the experiments on measuring the signals from other components of the LFA strip, the strip was incrementally moved within the coil.

### 2.7. Data Analysis

All experiments were performed in triplicates. Data points on graphs and bar charts represent the mean values, and error bars indicate the standard deviation from the mean (n = 3).

## 3. Results and Discussion

### 3.1. Scheme of Lateral Flow Assay Based on Magnetic Bioconjugates for CYFRA 21-1 Detection

For the CYFRA 21-1 detection, we used a lateral flow assay based on a sandwich immunoassay with magnetic bioconjugates as detectable labels (Figure 1a). First, MBCs were prepared using COOH-modified magnetic particles and an anti-CYFRA 21-1 antibody covalently immobilized via the carbodiimide method (see Section 2.2). This antibody bound to one of the epitopes of the CYFRA 21-1 molecule. The test strip analytical membrane contained a test line, where an antibody specific to another epitope of CYFRA 21-1 was immobilized, and a control line containing an anti-mouse antibody. The strip also included a conjugate pad with pre-dispensed and dried MBCs, as well as an absorbent pad that provided continuous fluid flow along the strip under capillary action.

During the assay, the sample is added to the strip, and the dried MBCs dissolve. If CYFRA 21-1 is present in the sample, the antibodies on the MBCs bind to it, forming complexes. These complexes migrate along the strip and are captured at the test line via interaction with the immobilized antibodies. The control line is formed when the antibodies on the MBCs interact with anti-mouse antibodies.

To obtain quantitative assay results, we employed both static and dynamic optical detection methods, as well as an electronic detection method using MPQ magnetometry (Figure 1b–d).

### 3.2. Kinetic Characterization of Antibodies

**Figure 2:**
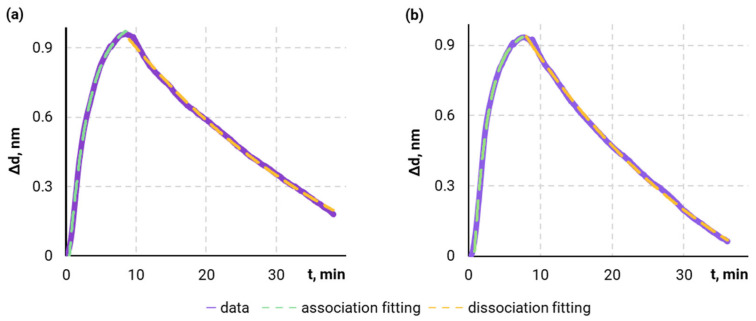
Experimental SPI sensorgrams for determining the kinetic parameters of interactions between CYFRA 21-1 antigen and monoclonal antibodies of XC42 (**a**) and XC10 (**b**) clones: the rising parts of the curves represent the association, while the falling parts correspond to the dissociation of the complexes.

The first series of experiments was dedicated to the kinetic characterization of the interaction between antibodies (mAbs, clones XC10 and XC42) and the CYFRA 21-1 molecules to determine key kinetic parameters such as the association rate constant (k_on_), dissociation rate constant (k_off_), and the corresponding equilibrium constants K_A_ and K_D_. These parameters largely govern the sensitivity of the developed lateral flow immunoassay system. The characterization was conducted using the SPI method (Section 2.3), which allowed real-time monitoring of interaction between mAbs and the CYFRA 21-1 antigen. The antibodies were immobilized on the glass surface of an SPI chip, and CYFRA 21-1 at various concentrations served as the analyte. The processes of association and dissociation of antibody–antigen complexes were recorded to plot the kinetic sensorgrams (Figure 2).

Based on these data, the kinetic interaction parameters were calculated (Table 1). The obtained high k_on_ values and low k_off_ values for both clones indicate high affinity of the antibodies to CYFRA 21-1. Notably, XC10 demonstrates higher affinity compared to XC42: although the k_on_ values are practically identical, the k_off_ values differ, with XC10 exhibiting a lower k_off_, providing higher stability of the “mAb XC10–CYFRA 21-1” complex. Due to its higher stability, mAb XC10 is preferable for immobilization on the test line of the analytical membrane, where it is essential to rapidly capture and retain the relatively large ‘sandwich’ complex of the analyte and magnetic bioconjugate throughout the assay. Consequently, mAb XC42 was chosen for immobilization on the surface of magnetic particles.

### 3.3. Optimization of Lateral Flow Immunoassay Conditions

**Figure 3:**
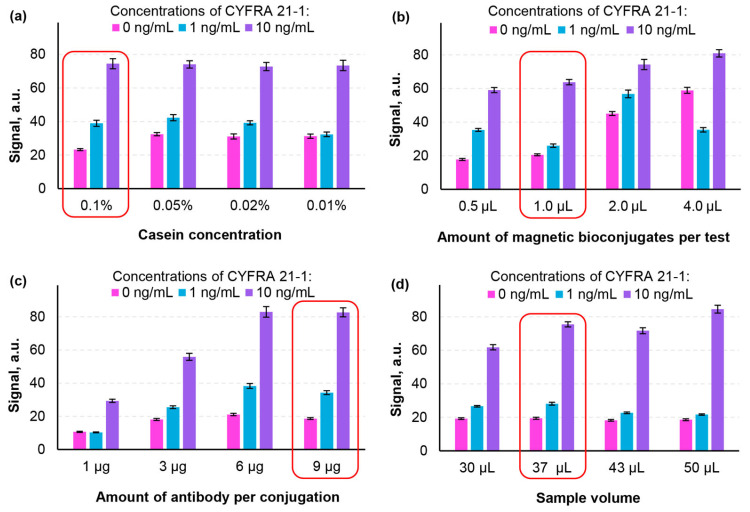
Optimization of assay conditions for the lateral flow immunoassay: effects of (**a**) casein concentration in the running buffer, (**b**) amount of magnetic bioconjugates per test, (**c**) amount of antibodies used for conjugation to magnetic particles, and (**d**) sample volume. The legend represents the concentrations of CYFRA 21-1 tumor marker in the probe (0, 1, and 10 ng/mL). The red boxes indicate the selected optimal conditions.

The next stage of the experiments aimed at optimization of the parameters of the MBC-based LFA to achieve maximum analytical sensitivity (characterized by the slope of the calibration curve) within the medically relevant concentration range of CYFRA 21-1 (1–10 ng/mL). First, the parameters such as casein concentration in the running buffer, the amount of antibodies conjugated to the magnetic particles, and the quantity of MBCs per test were optimized. The optimization criterion was the difference in optical signals recorded on the LFA test line when analyzing samples with relatively low (1 ng/mL) and high (10 ng/mL) concentrations of CYFRA 21-1. The optimization process was conducted step by step, and the results are presented in Figure 3a–c.

A 0.1% (*w*/*w*) concentration of casein was selected, as it provided a highest variation in signals between 1 ng/mL and 10 ng/mL concentrations without causing aggregation of MBCs (Figure 3a). Similarly, 1.0 μg of MBCs per test was chosen because higher amounts led to instability, while lower amounts reduced the signal difference (Figure 3b). The optimal antibody amount of 9 μg for conjugation to magnetic particles was determined based on the recorded signals (Figure 3c).

To select the optimal sample volume, the dynamics of optical signal formation on the test line was investigated. During the lateral flow assay, a video of the test line formation was recorded using a smartphone (see Section 2.5). The average intensity over time of the test line was then analyzed to determine the moment of signal saturation. As expected, an increase in the sample volume resulted in longer assay times, while decreasing the volume compromised sensitivity (Figure 3d). The objective of this optimization was to identify the conditions under which the assay time did not exceed 20 min. Based on the dynamics of signal variations, an assay time of 19 min was achieved with the sample volume of 37 μL.

An important advantage of magnetic bioconjugates over standard gold nanoparticles is the ability of non-destructive monitoring of their distribution within the LFA test strip using the MPQ method. In the following experiments, we demonstrate how this approach can be utilized to optimize assay conditions, particularly in selecting the appropriate buffer for drying MBCs on the conjugate pad. Colloidal solutions of MBCs were prepared in buffers containing sucrose at the following concentrations (*w*/*w*): 8.3%, 16.7%, 25%, 33.3%, 41.7%, 50%, 58.3%, and 66.7%. Sucrose not only acts as a stabilizer that facilitates the resuspension of biomolecules and MBCs from the fiberglass conjugate pad but also regulates assay time. The LFA strips were used to analyze samples containing 5 ng/mL of CYFRA 21-1 under optimal conditions. It was observed that the signal from the conjugate pad decreased as sucrose concentration increased. Importantly, the MPQ method has enabled the precise quantification of MBCs remaining on the conjugate pad—a parameter that visual and optical methods cannot reliably assess. The assay time was measured by recording signal dynamics, with the point of highest test line intensity that indicated the assay completion. Based on the magnetometry data, a buffer containing 50% sucrose was selected, as it allowed for an assay time of under 20 min (Figure S1, Supplementary Video S1).

### 3.4. Analytical Characteristics Achieved Using Magnetic and Optical Detection

**Figure 4:**
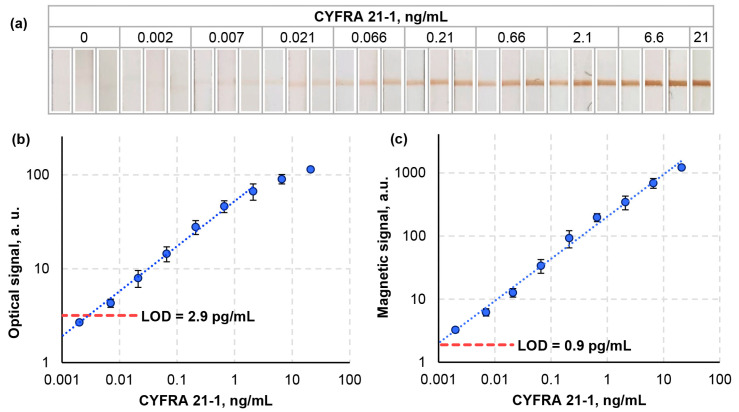
Calibration curves for CYFRA 21-1 determination using the lateral flow assay with visual (**a**), colorimetric (**b**), and MPQ (**c**) detection. The dashed line in plots (**b**,**c**) is intended to determine the limit of detection and corresponds to the negative control signal plus 3 of its standard deviations.

The next stage involved determining the analytical characteristics of the optimized lateral flow assay using MBCs. Samples with known concentrations of CYFRA 21-1 (0; 0.002; 0.007; 0.021; 0.066; 0.21; 0.66; 2.1; 6.6; and 21 ng/mL) were analyzed. Signals from the test lines were recorded by both optical and MPQ methods (Figure 4). Based on the calibration curves generated from these data, the limits of detection were calculated using the criterion of three standard deviations along with the dynamic range of detectable concentrations and the log-log sensitivity represented by the slope of calibration curve in logarithmic scales.

The static colorimetric detection results showed that the developed LFA achieved a limit of detection (LOD) of 2.9 pg/mL for CYFRA 21-1, a dynamic range of 3.9 orders of magnitude, and a sensitivity of 0.41. This indicates that the optical signal increases, on average, by 2.5 times with each tenfold increase in concentration. As shown in Table 2, these analytical characteristics surpass those reported in previous studies on LFA tests for CYFRA 21-1 detection [60,61,62,63,64]. This improvement can be attributed to the introduction of new quantitative methods during assay optimization, as well as to the highly efficient magnetic bioconjugates (MBCs). Notably, the MBCs used provided high optical contrast in the visible range.

Optical detection offers several advantages, including high sensitivity and the potential for rapid analysis using easily accessible tools like smartphones. Additionally, the option of qualitative (yes/no) visual readings of the test line makes the assay a useful tool for rapid point-of-care diagnostics. As seen in Figure 4a, the developed LFA enables visual interpretation with LOD of approximately 21 pg/mL, which is within the clinically relevant concentration range for CYFRA 21-1.

However, optical detection has limitations. Figure 4b shows that at higher analyte concentrations, the signal saturates, and that leads to deviations from the linear calibration curve. This effect is particularly due to the Beer–Lambert law, which confines the linearity of optical absorption at higher concentrations. Furthermore, the dynamic range of detectable signal changes is relatively limited. It can be seen that the optical signals correspondent to the LOD concentration (2.9 pg/mL) and to the maximum CYFRA 21-1 concentration of 21 ng/mL differ by a factor of only 39.6. Additionally, such factors as unstable lighting during optical detection, subjective visual interpretation, and changes in the optical properties of the analytical membrane upon drying may affect the accuracy of quantitative tests.

Using the MPQ detection, the developed magnetic LFA achieved a limit of detection of 0.9 pg/mL, a dynamic range spanning 4.4 orders of magnitude, and a sensitivity of 0.66. This means that the magnetic signal grows by an average of 4.5 times with each tenfold increase in concentration. As shown in Table 2, these values represent the record-breaking performance for LFA tests. Remarkably, no signal saturation was observed, and the calibration curve remained linear across the entire concentration range (Figure 4c). That was primarily because the magnetic detection was conducted throughout the entire thickness of the membrane rather than just at its surface. The higher sensitivity (greater slope of the calibration curve) and the fact that the signal changes 735-fold across the concentration range enable precise quantitative results, as errors in the signal detection have a smaller impact on the accuracy of concentration determination. Thus, the magnetic detection overcomes the limitations of optical detection, enhancing the assay’s quantitative capabilities.

The specificity of the developed method was further evaluated by adding to the samples alternative high-molecular-weight tumor markers at 10 ng/mL, including carcinoembryonic antigen (CEA), cancer antigen CA125, alpha-fetoprotein (AFP), and free prostate-specific antigen (fPSA). It was confirmed that the presence of these side molecules did not affect the registered signal (Figure S2). This demonstrates the high specificity of the method and the absence of cross-reactivity with the tested substances.

The method’s performance was also verified in human serum and artificial saliva. Two calibrated human serum samples were used: one containing CYFRA 21-1 at a concentration of 18.38 ng/mL and another devoid of CYFRA 21-1. These samples were mixed in various proportions and analyzed using the developed LFA test with the MPQ detection. The measured concentrations were determined from the calibration curve (Figure 4), and the recovery rates were calculated as the ratio of the measured concentration to the expected concentration (Table 3). In artificial saliva, a spike-and-recovery test was conducted by adding known concentrations of CYFRA 21-1 (0, 1, 3, and 10 ng/mL). The recovery rates ranged from 84% to 124%, which indicated the method’s reliability in both serum and saliva matrices.

## 4. Conclusions

This study presents the successful development of a highly sensitive and specific lateral flow assay for detection of the CYFRA 21-1 tumor marker based on using magnetic bioconjugates along with both optical and magnetic registration methods. By integrating advanced methods such as spectral-phase interferometry, static and dynamic colorimetric detection, as well as magnetic particle quantification, we provide a framework for optimizing the assay conditions to improve its performance.

The comparative evaluation of MPQ-based and colorimetry-based detection techniques demonstrates that the magnetic detection can overcome several limitations inherent to conventional approaches in LFA that often hinder accurate quantification. The absence of signal saturation, significantly improved (by more than three times) limit of detection, higher sensitivity (reflected in the calibration curve slope), and much wider dynamic range of detected signals (735-fold for magnetic detection and 39.6-fold for optical detection) underscore the potential of this test system to achieve reliable quantitative measurements.

The advantages of the proposed approach, including rapid turnaround and adaptability to resource-limited settings, make it a promising candidate for widespread use in point-of-care diagnostics, particularly in underserved areas, as well as for non-invasive saliva testing. The obtained results open up possibilities to investigate its applicability to other biomarkers and possibilities of multiplexing capabilities for the simultaneous detection of multiple biomarkers, thereby enhancing the diagnostic efficiency and providing a more comprehensive assessment of the disease status.

## Figures and Tables

**Table 1:** Kinetic properties of CYFRA 21-1 antibodies obtained by SPI method.

Clone	k_on_, M^−1^c^−1^	k_off_, c^−1^	K_d_, M	K_a_, M^−1^
XC10	(1.4 ± 0.2) × 10^5^	(3.9 ± 0.4) × 10^−4^	2.86 × 10^−9^	3.50 × 10^8^
XC42	(1.3 ± 0.1) × 10^5^	(7.1 ± 0.5) × 10^−4^	5.40 × 10^−9^	1.85 × 10^8^

**Table 2:** Comparison of developed MBC-based LFA with alternative LFA-based approaches.

Method	Limit of Detection, ng/mL	Dynamic Range, Orders	Reference
Fluorescent nanosphere-based immunochromatographic test strip	0.071	1.81	[60]
Colloidal gold immunochromatographic test strip	0.55	2.96	[61]
CdTe/CdSe hydrophobic quantum dots-based lateral flow assay	0.16	2.57	[62]
Trimodal lateral flow assay (magnetic, colorimetric, and fluorescent)	0.26	1.11	[63]
NIR-Ⅱ fluorescence lateral flow immunoassay platform	0.18	2.74	[64]
MBC-based LFA with optical detection	0.0029	3.9	This work
MBC-based LFA with magnetic detection	0.0009	4.4	This work

**Table 3:** Recovery rates of CYFRA 21-1 in serum and artificial saliva matrices.

Matrix	Expected (ng/mL)	Measured (ng/mL)	Recovery (%)
Serum	0	N/A	N/A
Serum	0.57	0.48	84%
Serum	1.15	1.16	101%
Serum	2.30	2.14	93%
Serum	4.60	3.95	86%
Serum	9.19	9.65	105%
Serum	18.38	17.09	93%
Artificial Saliva	0	N/A	N/A
Artificial Saliva	1	1.24	124%
Artificial Saliva	3	3.36	112%
Artificial Saliva	10	11.81	118%

## Data Availability

Data are contained within the article.

## Supplementary Materials

The following supporting information can be downloaded at: https://www.mdpi.com/article/10.3390/bios14120607/s1, Figure S1: Magnetic bioconjugates (MBCs) optimization with sucrose buffers at different concentrations; Figure S2: Specificity assessment of the assay in the presence of alternative tumor markers; Supplementary Video S1: Signal change dynamics registration at different concentrations of sucrose in drying buffer.
