# Supplementary material for: Enhanced Analytical Performance in CYFRA 21-1 Detection Using Lateral Flow Assay with Magnetic Bioconjugates: Integration and Comparison of Magnetic and Optical Registration
Source: Biosensors (Basel). 2024 Dec 11;14(12):607. doi: 10.3390/bios14120607 (PMC11674727; doi:10.3390/bios14120607)
Supplement: Supplementary file 1 [file biosensors-14-00607-s001.zip › biosensors-3286167-supplementary.pdf]

Supplementary Materials

Enhanced analytical performance in CYFRA 21-1 detection using lateral flow assay with magnetic bioconjugates: integration and comparison of magnetic and optical registration

Artemiy M. Skirda [1,†], Alexey V. Orlov [1,†,*], Juri A. Malkerov [1,2,†], Sergey L. Znoyko [1], Alexandra S. Rakitina [1], and Petr I. Nikitin [1,2*]

[1] Prokhorov General Physics Institute of the Russian Academy of Sciences, 38 Vavilov Street, 119991 Moscow, Russia
[2] National Research Nuclear University MEPhI (Moscow Engineering Physics Institute), 31 Kashirskoe Shosse, 115409 Moscow, Russia
[*] Correspondence: alexey.orlov@kapella.gpi.ru (A.V.O.); nikitin@kapella.gpi.ru (P.I.N.)
[†] These authors contributed equally to this work.

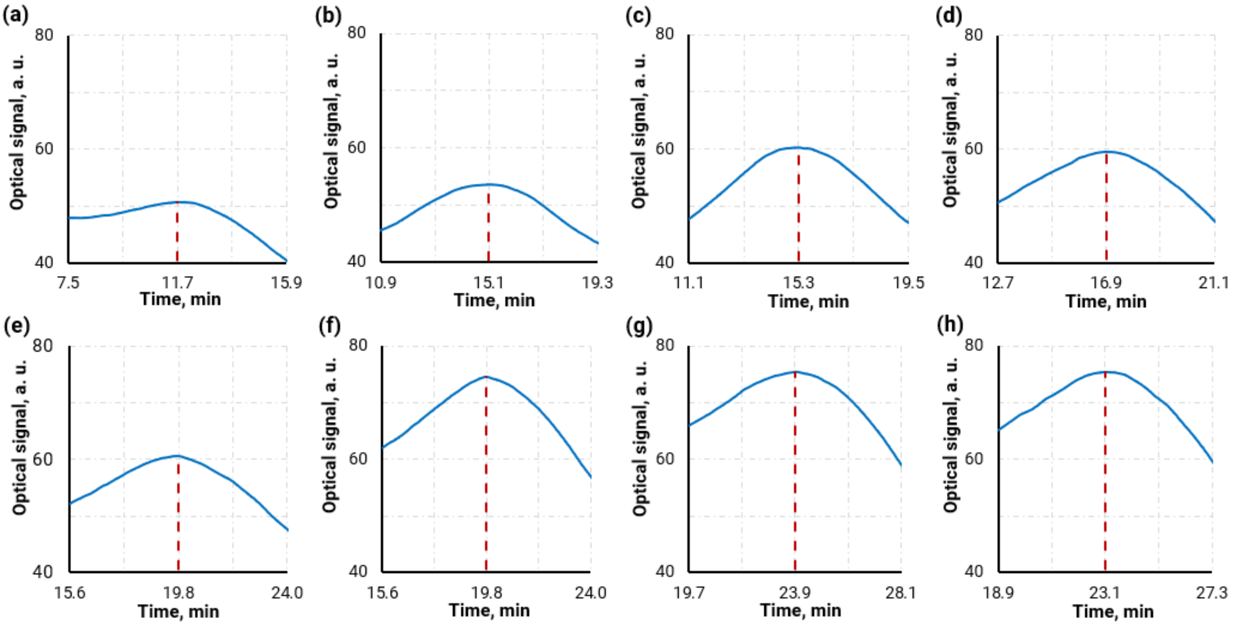

Figure S1. Optical registration of signal change dynamics for test strips with sugar contents in drying buffer equal to a) 8.3% b) 16.7% c) 25% d) 33.3% e) 41.7% f) 50% g) 58.3% and h) 66.7%. The red dashed line indicates the point of maximum intensity level reached at the end of the assay.

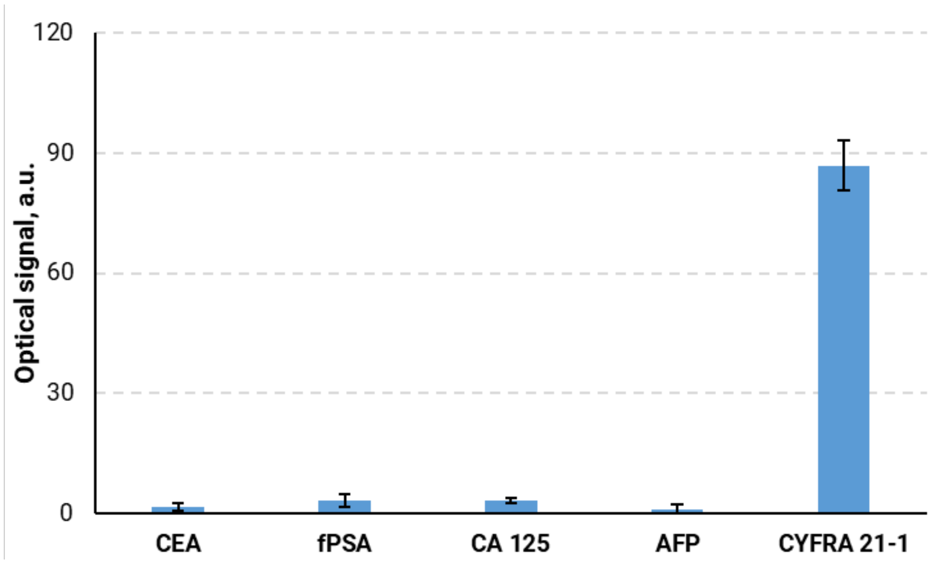

Figure S2. Specificity assessment of the assay in the presence of alternative tumor markers. All the tested probes contained corresponding tumor markers dissolved in running buffer at a 10 ng/ml concentration.
